# The use of large animals to facilitate the process of MSC going from laboratory to patient—‘bench to bedside’

**DOI:** 10.1007/s10565-020-09521-9

**Published:** 2020-03-23

**Authors:** W. E. Hotham, F. M. D. Henson

**Affiliations:** 1 Division of Trauma and Orthopaedic Surgery, Cambridge University, Cambridge, UK; 2 Animal Health Trust, Newmarket, UK

**Keywords:** Mesenchymal stem cell, Large animal, Osteoarthritis, Myocardial infarction

## Abstract

Large animal models have been widely used to facilitate the translation of mesenchymal stem cells (MSC) from the laboratory to patient. MSC, with their multi-potent capacity, have been proposed to have therapeutic benefits in a number of pathological conditions. Laboratory studies allow the investigation of cellular and molecular interactions, while small animal models allow initial ‘proof of concept’ experiments. Large animals (dogs, pigs, sheep, goats and horses) are more similar physiologically and structurally to man. These models have allowed clinically relevant assessments of safety, efficacy and dosing of different MSC sources prior to clinical trials. In this review, we recapitulate the use of large animal models to facilitate the use of MSC to treat myocardial infarction—an example of one large animal model being considered the ‘gold standard’ for research and osteoarthritis—an example of the complexities of using different large animal models in a multifactorial disease. These examples show how large animals can provide a research platform that can be used to evaluate the value of cell-based therapies and facilitate the process of ‘bench to bedside’.

## Introduction

Animals are used in research where there is a need to study the effect of a treatment on a whole tissue or living organism (Barré-Sinoussi and Montagutelli 2015). Humans and animals share many similarities both morphologically and pathologically and animas are regularly used to study disease onset, progression and treatment (Solinas et al. 2014). In the development of novel therapeutics, animal models can also provide vital information on safety and efficacy prior to human studies (Bianco et al. 2013). All animal research is tightly regulated by the country in which it is being undertaken and research on animals within the EU is regulated under Directive 2010/63/EU (Macrì et al. 2013). This directive was established in all EU states in 2013 to ensure a harmoniously high standard of animal research (Macrì et al. 2013). The directive ensures a contentious effort to implement strategies to reduce the number of animals used in research while refining techniques to reduce predicted pain, suffering, distress and/or lasting pain whilst also improving animal husbandry. Animal experiments are conducted on a wide variety of species including invertebrates, fish, birds and mammals (with mammalian species being divided into ‘small’ animal or ‘large’ animal models).

An animal is considered a ‘large animal’ when the species in question is non rodent, rabbit or guinea pig (Thomas et al. 2012). The more commonly used large animal models in research include horses, cows, pigs, sheep, goats, primates and dogs, and the choice of animal model depends on multiple factors, including the type of experiment, its duration, husbandry costs, handling logistics and measurement parameters (Kuyinu et al. 2016).

Whilst small animals have been invaluable in furthering modern understanding of disease by providing an opportunity to conduct research cheaply, rapidly and with a degree of complexity not offered by in vitro experiments or other species, in some situations the information that can be provided by large animals is required to answer specific research questions (Moran et al. 2016; Ziegler et al. 2016). Large animal models offer advantages over small animal models in many areas. They are more similar physiologically and anatomically to man (size, tissue structure and life span) and large animals are an ‘out bred’ population that more closely represents the heterogeneity of the human population than the ‘inbred’ small animal strains used in research (Salvatore et al. 2008). Large animals are phylogenetically closer to humans than rodents and therefore, at a molecular level, they have greater sequence homology with humans making interpretation of molecular events in large animals more relevant to man (Henze and Urban 2010). Practically, the consequence of working with a large animal means that more body fluids and cells can be collected with which to perform experiments.

To illustrate how using large animals have facilitated the process of moving MSC from ‘bench to bedside’, two examples will be considered in this review—the treatment of myocardial infarction (MI) and osteoarthritis (OA). The former represents an example of one single large animal model being considered the ‘gold standard’ for research, while the latter is an example of the complexities of using large animal models in a multifactorial disease.

## Large animals models for treating myocardial disease using MSC

There has been a recent increase in the incidence of MI worldwide (Rumana et al. 2008). This is due to many factors such as an ageing population, more sedentary lifestyles and generally poorer diets (Mohseni et al. 2017). MI is diagnosed as a cessation of correct blood flow to the heart, leading, in clinical practice, to sudden death, or ischaemia and subsequent loss of cardiomyocytes (Chiong et al. 2011; Reddy 2015). The chances of surviving one MI are high, but post MI complications are of clinical significance (Chiong et al. 2011). Localised myocardium loss leads to heart wall thinning and ventricle dysfunction (Lu et al. 2015). In order to maintain heart function, the left ventricle dilates to maintain stroke volume and cardiac output (Mohseni et al. 2017). However, left ventricular dilatation leads to heart failure and eventual death and MI clearly represents a key pathology that requires therapy (Reddy 2015). Over the past 40 years, our understanding of MI has increased and, with this, so have the number of MI related publications (Saleh and Ambrose 2018).

The possibility of using MSC to regenerate cardiomyocytes became possible when it was demonstrated in vitro that, in addition to the well-recognised differentiation products of MSC (into osteoblasts, adipocytes and chondrocytes), MSC can be differentiated into cardiac cell types (White et al. 2016; Szaraz et al. 2017; Guo et al. 2018). For example, Szaraz et al. (2017) differentiated human umbilical MSC into ‘cardiac like cells’ that expressed cardiac myocyte differentiation markers such as myocyte enhanced factor 2C, cardiac troponin T, heavy chain cardiac myosin, signal regulatory protein α and connexion 43. Similarly, Markmee et al. (2017) showed that after 21 days in cardiogenic culture medium, MSC displayed the cardiomyocyte markers GATA binding protein 4, cardiac muscle troponin, connexin 43 and Nkx2.5. Cross-talk between MSC and cardiomyocytes was demonstrated by Gao et al. (2016) who showed that co-culture of MSC with neonatal rat ventricular myocytes lead to the development of partial electrical properties similar to the cardiomyocytes (Gao et al. 2016).

In addition to the ability of MSC to differentiate into ‘cardiac-like cells’, it has also been shown that MSC can support cardiac cell viability via secreted factors. Ismail et al. (2014) created a model of hydrogen peroxide-induced cardiomyocyte injury and showed that neonatal cardiomyocytes and the cardiac myoblast cell line H9c2 both had significantly increased viability and reduced apoptosis in the presence of MSC secreted SC1 (Ismail et al. 2014). Xiang et al. (2009) also showed that the application of MSC conditioned media to neonatal rat cardiomyocytes and reduced cardiomyocyte apoptosis via effects on the mitochondrial pathway (Xiang et al. 2009).

Following these encouraging in vitro results, subsequent small animal studies showed that MSC had therapeutic efficacy in a MI model. Functionally, MSC were shown to have a number of positive effects including improving left ventricle function, increasing vascular density, decreasing scar size (López et al. 2013; Wang et al. 2018), left ventricle stroke volumes and ejection fractions (Dai et al. 2005) and increasing remodelling of gap junctions (Dai et al. 2005; López et al. 2013; Wang et al. 2018). There is also some evidence that MSC differentiate, in situ, into cardiac cells at sites of damage (Nagaya et al. 2005).

However, whilst small animal studies have been useful to show proof of concept for the use of MSC to treat MI, it has been necessary to use large animal models, specifically the porcine ischaemic MI model, to confirm the suitability of this cell therapy in man. Small animal cardiac parameters such as heart rate, coronary architecture and capillary density (Harding et al. 2013) are markedly different to man, whereas large animal hearts are more similar (Harding et al. 2013). The porcine model is the most used for MI research due to the similarities in heart size and coronary anatomy between pigs and humans (Swindle et al. 2012). Also, again on a practical note, the relatively high sequence homology between porcine and human proteins more readily facilitates research enabling commercially purchased reagents to be used (Dreher et al. 2011).

The ‘gold standard’ model of porcine MI that is used in all published papers is the artery occlusion model, in which, a dilation catheter is inflated in the coronary artery. This catheter blocks blood flow to part of the heart causing infarction development. However, the remainder of the heart will continue to receive normal blood perfusion and thus provides a defined border zone between normal and damaged tissue for comparative evaluation (McCall et al. 2012). Schuleri et al. (2009) showed a positive effect of using autologous BM-MSC, administered 12 weeks post infarct to treat MI. Magnet resonance imaging (MRI) was used to assess infarct size, myocardial blood flow and left ventricle function. In this study, an apparent dose-dependent effect of MSC administration on infarct size was observed.

Whilst Schuleri et al. (2009) used autologous MSC in their experimental work, there is much interest in allogeneic MSC therapy. Allogeneic MSC offer significant advantages over autologous MSC including their ease of use, reduced cost and absence of donor site complications (Schuleri et al. 2009). Quevedo et al. (2009) showed that allogeneic MSC are able to regenerate an experimentally created, chronically infarcted myocardium via long-term engraftment (Quevedo et al. 2009). Following MRI, cell fate was confirmed using Y chromosome cell tracking. In comparison to the control group, infarct size reduced by 5.4%, ejection fraction increased by 6.3% and levels of MSC engraftment correlated with functional recovery levels (measured by assessing contractility and myocardial blood flow). In this study, the implanted MSC were only detected within the infarct area or the infarct border with 14% showing evidence of myocyte commitment (assessed by the presence of cardiac transcription factors GATA-4 and Nkx2.5 or structural cardiac proteins α-sarcomeric actin and tropomyosin) (Quevedo et al. 2009). Similarly Williams et al. (2013) also investigated the use of allogeneic MSC with excellent results—a 19.62% reduction in scar size after 12 weeks, progressing to 28.09% after 24 weeks and a functional improvement in heart function (Williams et al. 2013).

The studies reported above all showed positive effect of administrating MSC as early as 12 weeks post infarct creation. However, administration at earlier time points has also been shown to be efficacious, for example, administration at 3 days post infarct (Hatzistergos et al., 2010), suggesting that the optimal time window for therapeutic intervention is not fully established. Lee et al. showed that administering EVs after 30 min post infarct had no effect, thus work continues in the porcine model to determine these important criteria. Examples of these studies are summarised in Table 1.

**Table 1:** Examples of the different cell types used and when they were administered in large animal models using MSC as a therapeutic for myocardial infarction

Cell type	Cell source	Cell number × 10^6^	Administration date post infarct	Outcome	Author and date
BM-MSC	Autologous	20	14 days	Decreased infarct size, improved left ventricle function and myocardial blood flow	Schuleri et al. 2009
	Allogeneic	200	12 weeks	Decreased infarct size, increased ejection fraction, MSC engraftment and differentiation into cardiac like cells	Quevedo et al. 2009
A-MSC	Autologous	2	30 min	No effect on left ventricle ejection fraction, improved blood perfusion in the defect	Lee et al. 2015
	Allogeneic	214	9 days	Angiogenesis, vasculogenesis, decreased fibrosis and cardiac hypertrophy	Mazo et al. 2012
UC-MSC	Autologous	No examples were found in the literature			
	Allogeneic	1.5 × 10^6^/kg of body weight	8 weeks	Improved left ventricle infarct area but no effect on perfusion, reduced fibrosis and inflammation	Lim et al. 2018

*BM*-*MSC* bone marrow mesenchymal stem cells, *A*-*MSC* adipose mesenchymal stem cells, *UC*-*MSC* umbilical cord mesenchymal stem cells

Due to positive results in the porcine MI model, MSC are now being used in clinical trials to treat a variety of cardiac diseases in man (Table 2). In these clinical trials to date, all have reported that the use of MSC is safe and a significant majority of studies have reported a positive outcome despite a high number of variables in the studies. However, it should be noted that many knowledge gaps still exist and study designs should now attempt to gain knowledge, such as the optimum dosage, cell source and time of injection.

**Table 2:** Published clinical trials that use defined numbers of mesenchymal stem cells (MSC) for treating heart disease

Author and date	Type of heart disease	MSC type/source	Number of cells administered ×10^6^	Study type	Outcome
Ascheim et al. 2014	ICM or NICM	BM, allogeneic	25	Phase 2	Safe and positive
Bartolucci et al. 2017	ICM or NICM	US, allogeneic	1/kg of body weight	Phase 1/2	Safe and positive
Bartunek et al. 2013	ICM	BM, autologous	6–12 after treatment with cardiac cocktail	Phase 2/3	Safe and positive
Bartunek et al. 2017	ICM	BM, autologous	24	Phase 3	Safe and positive
Butler et al. 2017	NICM	BM, allogeneic	1.5/kg body weight	Phase 2	Safe and positive
Chen et al. 2004	AMI	BM, autologous	50 to 60	Phase 2	Safe and positive
Chen et al. 2006	ICM	BM, autologous	> 5	Phase 1/2	Safe and positive
Florea et al. 2017	ICM	BM, allogeneic	20 or 100	Phase 2	Safe and positive
Gao et al. 2015	AMI	UC, allogeneic	6	Phase 2	Safe and positive
Guijarro et al. 2016	ICM	BM, autologous	61	Phase 1	Safe
Hare et al. 2009	AMI	BM, allogeneic	0.5, 1.6 and 5/kg	Phase 1	Safe
Hare et al. 2012	ICM	BM, allogeneic and autologous	20, 100 or 200	Phase 1/2	Safe and positive
Hare et al. 2017	DCM	BM, autologous	20, 100 or 200	Phase 1/2	Safe and positive
Henry et al. 2017	ICM	ABM, autologous	40 and 80	Phase 2	Safe and positive
Houtgraaf et al. 2012	AMI	ABM, autologous	20	Phase 1/2	Safe and positive
Karantalis et al. 2014	ICM	BM, autologous	8–20	Phase 2/3	Safe and positive
Kastrup et al. 2017	ICM	ABM, allogeneic	110	Phase 1	Safe
Mathiasen et al. 2015	ICM	BM, autologous	77.5	Phase 1/2	Safe and positive
Mohamadnejad et al. 2007	ICM	BM, autologous	32	Phase 1	Safe
Musialek et al. 2015	AMI	UC, allogeneic	30	Phase 1	Safe
Qayyum et al. 2017	ICM	ABM, autologous	70	Phase 2	Safe and positive
Rodrigo et al. 2013	AMI	BM, autologous	10	Phase 1	Safe

This table shows the type of heart disease treated, the source of the MSC, the cell number and the study outcomes. *ICM* ischemic cardiomyopathy, *NICM* non-ischemic cardiomyopathy, *AMI* acute myocardial infarction, *DCM* dilated cardiomyopathy, *BM* bone marrow derived MSC, *UC* umbilical cord derived MSC, *ABM* adipose derived MSC

## Large animal models for osteoarthritis

In contrast to the single porcine large animal model that has been used to show the efficacy of MSC in the treatment of MI, a variety of large animal models have been used to demonstrate the therapeutic benefits of MSC in the treatment of Osteoarthritis (OA) prior to clinical trials.

OA is the gradual degeneration of articular cartilage within synovial joints (Sharma et al. 2013). It is estimated that, worldwide, eight million people over the age of 65 suffer with this disease (Neogi 2013). OA is the result of structural and functional failures within the synovial joint (Nuki 1999). This is due to the pathological loss of articular cartilage coupled with sub-chondral bone thickening, osteophyte development, ligament degeneration and varying levels of inflammation (Chen et al. 2017). These pathologies all contribute to pain-induced joint morbidity (Chen et al. 2017). OA can be classified into primary and secondary forms based on aetiology. Primary forms of the disease are age-related, whilst trauma is the most common form of secondary OA (Samson et al. 2007).

There are currently no disease-modifying therapeutics licensed for use in OA and there is a huge clinical need for effective therapies. In recent years, MSC have been used to treat OA in pre-clinical and clinical studies. The rationale behind the use of MSC to treat OA was initially proposed to be harnessing the potential of MSC to differentiated into mesodermal tissues including cartilage. It was proposed that MSC, injected into damaged joints, differentiate into the tissues of the joints and healed the lesions. However, more mature understanding of the mechanism of action of MSC suggest that rather than acting as building blocks, they are acting in a paracrine fashion to modulate cellular responses (Kong et al. 2017).

As outlined for MI research above, the pathway to human clinical trials for using MSC as an OA therapeutic is based on in vitro, small animal and then large animal models.

Evidence that MSC have a beneficial effect on the native cells within the joint has been shown in numerous studies (reviewed by (Li et al. 2019). For example, the co-culture of chondrocytes and MSC has been shown to increase glycosaminoglycan synthetic activity as well as increased expression of chondrogenesis-related genes (type II collagen and SOX-9) whilst simultaneously downregulating the expression of osteogenic markers and chondrocyte hypertrophic markers (Bian et al. 2011; Huang et al. 2018; Kim et al. 2018). Similarly, it has been shown that MSC can promote both macroscopic and microscopic healing of meniscal defects, usually in the presence of biocompatible scaffolds (Pabbruwe et al. 2010; Zellner et al. 2010; Mandal et al. 2011; Nerurkar et al. 2011).

In small animals, MSC have been shown to have disease-modifying properties in a number of experimental small OA models, such as in mouse and rabbit anterior cruciate ligament transection models (Chiang et al. 2016). Similarly, Tang et al. (2017) also showed that MSC decreased osteophyte and fibrous tissue formation and increased type II collagen and aggrecan in a rat medial menisectomy model after the administration of MSC (Tang et al. 2017). Improved cartilage repair has also been shown in chemically induced murine arthritis models and in focal cartilage defect models (Kehoe et al. 2014; Mak et al. 2016).

Whilst MSC have been used in small animal OA models as described above, large animals offer significant advantages over small animals for the assessment of the therapeutic benefits of MSC prior to clinical trials. Large animals have similar bone development to man compared to small animals, i.e. they have closed growth plates at skeletal maturity and large animal models of OA occur more slowly than in small animal models, mimicking the natural disease in man (McGovern et al. 2018). However, it must be noted that whilst all large animals will develop OA naturally as they age, there are no models of spontaneous early onset OA as there are in small animals (Bendele et al. 1989; Jimenez et al. 1997; Poole et al. 2010).

Unlike the use of a single ‘gold standard’ large animal model for evaluating the effects of MSC in MI, many models exist for the generation of OA in large animals. Experimental models of large animal OA are primarily surgically induced damage, although there are two reports of the use of MSC to treat chemically induced arthritis. Mokbel et al. (2011) used amphotericin-B in a donkey OA induction model and demonstrated that the injected cells had integrated within the existing cartilage and the reparative effects of the MSC were observed both clinically and radiographically (Mokbel et al. 2011)*.* Barrachina et al. (2018) described the use of bone marrow MSC to treat amphotericin-B induced arthritis in an equine radio-carpal joint. In this study, the application of MSC decreased synovial inflammation, enhanced the gross appearance of the cartilage and delayed proteoglycan loss in comparison to the control. This study also reported differences in outcome between naïve MSC and MSC primed with tumour necrosis factor—alpha (TNFα) and interferon-gamma (IFN-γ). This data is particularly useful in considering the clinical translation of MSC as there is ongoing discussion as to the need for MSC priming/conditioning prior to use (Succar et al. 2016; Barrachina et al. 2018)

Whilst there are only currently two reported studies on the use of MSC to treat chemically induced arthritis in large animal models, many studies have reported the use of different MSC to treat surgically induced arthritis as a proxy for the human disease. (Table 3). In these studies a wide range of large animal species and different surgical techniques have been used to model OA. These techniques include anterior cruciate ligament transection (ACLT), meniscectomy and medial meniscal transection and osteochondral fragment defect models. These are all well-standardised procedures, with each model posing its own advantages and disadvantages (reviewed in (Kuyinu et al. 2016).

**Table 3:** Examples of large animal models used to study the efficacy of bone marrow (BM), adipose (A), umbilical cord (UC) and synovial (S) derived MSC in the treatment of experimentally induced osteoarthritis (OA)

Animal	Cell type	Method of OA induction	Cell source	Outcome	Cell number ×10^6^	Author and date
Sheep	BM-MSC	ACLT + medial meniscectomy	Autologous	Meniscal and cartilage repair	10	Song et al. 2014
	A-MSC	ACLT + medial meniscectomy	Autologous	Cartilage repair	20 million	Ude et al. 2014
	UC- MSC	ACLT	Allogeneic	Cartilage repair	50 Million	Wang et al. 2009
	S-MSC	No examples were found in the literature				
Goat	BM-MSC	ACLT	Autologous	Cartilage repair	10 million	Murphy et al. 2003
	A-MSC	No examples were found in the literature				
	UC-MSC	No examples were found in the literature				
	S-MSC	No examples were found in the literature				
Pig	BM-MSC	No examples were found in the literature				
	A-MSC	Bilateral medial meniscectomy	Allogeneic	No significant repair but MSC located within the damaged tissue	10 million	Xia et al. 2018
	UC-MSC	No examples were found in the literature				
	S-MSC	No examples were found in the literature				
Horse	BM-MSC	Osteochondral fragmentation	Autologous	No significant results observed	16.3 million	Frisbie et al. 2009
	A- MSC					
	UC-MSC	No examples were found in the literature				
	S-MSC	No examples were found in the literature				
Donkey	BM-MSC	Partial thickness cartilage defect				
	A-MSC	Full thickness Cartilage defect	Autologous	Clinical and radiographic improvement	2 million	Mokbel et al. 2011
	UC-MSC	No examples were found in the literature				
	S-MSC	No examples were found in the literature				
Dog	BM-MSC	Full thickness cartilage defect	Autologous	Macroscopic and histological improvements following MSC administration with no adverse effects	10 million	Li et al., 2019
	A-MSC	Partial thickness cartilage defect	Allogeneic	Improvements in modified O’Driscoll histological score	5 million	Miki et al. 2015
	UC-MSC	Partial thickness cartilage defect	Allogeneic	Cartilage repair	15 million	Park et al. 2013
	S-MSC	No examples were found in the literature				

*ACLT* anterior cruciate ligament transection, *MSC* mesenchymal stem cells, *OA* osteoarthritis

Whilst many studies use autologous cells, as discussed previously in the treatment of MI, the use of allogeneic MSC to treat OA is of considerable interest. For example, human BM-MSC were used to treat ACLT induced OA in a porcine model 16 weeks post-surgery (Tseng et al. 2018). At 5 months post implantation, there was a significant difference between the regeneration of new tissue, with the treated group showing evidence of cartilage-like tissue. Similarly, Hatsushika et al.(2014) investigated the effect of allogeneic synovial MSC following partial meniscectomy in a porcine model and showed increased meniscus regeneration and prevention of OA progression by week 16 post-surgery (Hatsushika et al. 2014). Murphy et al. (2003) has also shown that the administration of allogeneic bone marrow MSC following ACTL in goats led to significantly increased tissue regeneration including the meniscus and decreased articular cartilage degeneration, osteophyte remodelling and subchondral sclerosis in comparison to the hyaluronan control(Murphy et al. 2003). These studies are important for the potential clinical applications of MSC as they may suggest there is no requirement for donor matching when using MSC therapeutically.

Whilst the studies above and those reported and summarised in Table 3 shows that MSC had a positive effect in a number of different models of OA, large animal studies have shown that MSC therapies are not always successful. Evaluation of the effects of allogeneic MSC on the development of OA following complete meniscectomy in a sheep model has been reported (Song et al. 2014; Delling et al. 2015). After 12 weeks, MRI, radiography and post-mortem evaluation showed no significant difference in the degree of OA between the treatment group and the control. Similarly, the use of MSC in the osteochondral fragment model of OA induction in horses showed no significant effects (Frisbie et al. 2009). This reporting of negative results from a large animal model is important data, inducing caution in the use of these cells. MSC therapy has widely been touted as a miraculous ‘cure all’, particularly in the popular press and amongst less scrupulous clinicians, and stringent efforts must continue to be made to ensure tight but feasible regulation of these therapies to ensure patient safety, as the use of MSC to treat patients is well underway (Table 4) (Bianco et al. 2013). A number of controlled clinical trials have been reported, with good outcomes in both visual analogue scale for chronic pain and western Ontario and McMaster Universities arthritis index scores (measures of joint morbidity), as well as range of movement, improved pain and joint motility scores following treatment (Lamo-Espinosa et al. 2016; Pers et al. 2016). These studies demonstrate the translation of MSC therapy into man whilst large animal therapeutic trials remain ongoing.

**Table 4:** Lists of the published clinical trials that use mesenchymal stem cells (MSC) for treating osteoarthritis (OA), the method of administration, the source of the MSC and the study outcomes

Author and date	Mode of delivery	MSC type and source	Phase	Outcome
Shapiro et al. 2017	Single intra-articular	Autologous bone marrow	1	Safe and positive
Chahal et al. 2019	Single intra-articular	Autologous bone marrow	1/2	Safe and positive
Emadedin et al. 2015	Single intra-articular	Autologous bone marrow	1	Safe
De Girolamo et al. 2010	Single intra-articular	Autologous haematopoietic stem cells from bone marrow	1	Safe
Gupta et al. 2016	Single intra-articular	Allogeneic bone marrow	2	Safe and positive
Lamo-Espinosa et al. 2018	Single intra-articular	Autologous bone marrow	1/2	Safe and positive
Matas et al. 2019	Single intra-articular	Allogeneic umbilical cord	1/2	Safe and positive
Al-Najar et al. 2017	Double intra-articular	Bone Marrow	2	Safe and positive
Orozco et al. 2013	Single intra-articular	Bone Marrow	1/2	Safe and positive
Ruane, 2019	Single intra-articular	Bone Marrow	2	Safe and positive
Shadmanfar et al., 2018	Single intra-articular	Bone Marrow	2/3	Safe and positive
Song et al. 2018	Single intra-articular	Adipose derived	1/2	Safe and positive
Soler et al. 2016	Single intra-articular	Bone marrow	1/2	Safe and positive
Taghiyar et al., 2010	Single intra-articular	Bone marrow	1	Safe

## Conclusions

Large animal models have been widely used to facilitate the translation of MSC from the laboratory to patient. The aim of this review is to illustrate how MSC have been translated to man through large animal models. For this, two very different examples have been used—MI (where one gold standard large animal model has been used in one species to show efficacy) and OA (where multiple species and models have been used). It is clear that using multiple models and different experimental approaches makes interpretation of results difficult and the use of a single large animal model is preferable. It is also clear that the majority of publications only report positive outcomes of MSC therapy and that encouragement of the publication of negative outcomes should be made as this will allow a more accurate assessment of therapeutic efficiency. However, used appropriately, large animal models allow clinically relevant assessments of safety, efficacy and dosing prior to clinical trials and continue to provide a research platform that can be used to evaluate the value of cell-based therapies.
